# Embryonic thermal manipulation: a potential strategy to mitigate heat stress in broiler chickens for sustainable poultry production

**DOI:** 10.1186/s40104-024-01028-1

**Published:** 2024-06-04

**Authors:** Sadid Al Amaz, Birendra Mishra

**Affiliations:** Department of Human Nutrition, Food and Animal Sciences, College of Tropical Agriculture and Human Resources, University of Hawaii at Manoa, AgSci 216, 1955 East-West Rd, Honolulu, HI 96822 USA

**Keywords:** Broiler, Embryo, Epigenetics, Global warming, Thermoregulation

## Abstract

Due to high environmental temperatures and climate change, heat stress is a severe concern for poultry health and production, increasing the propensity for food insecurity. With climate change causing higher temperatures and erratic weather patterns in recent years, poultry are increasingly vulnerable to this environmental stressor. To mitigate heat stress, nutritional, genetic, and managerial strategies have been implemented with some success. However, these strategies did not adequately and sustainably reduce the heat stress. Therefore, it is crucial to take proactive measures to mitigate the effects of heat stress on poultry, ensuring optimal production and promoting poultry well-being. Embryonic thermal manipulation (TM) involves manipulating the embryonic environment’s temperature to enhance broilers’ thermotolerance and growth performance. One of the most significant benefits of this approach is its cost-effectiveness and saving time associated with traditional management practices. Given its numerous advantages, embryonic TM  is a promising strategy for enhancing broiler production and profitability in the poultry industry. TM increases the standard incubation temperature in the mid or late embryonic stage to induce epigenetic thermal adaption and embryonic metabolism. Therefore, this review aims to summarize the available literature and scientific evidence of the beneficial effect of pre-hatch thermal manipulation on broiler health and performance.

## Introduction

Climate change has a threatening effect on the environment, animal production, and human health in the world. The Intergovernmental Panel on Climate Change reported that the environmental temperature had risen 1.53 ºC from 1850–1900 to 2006–2015 [1]. From 2011 to 2020, the average global surface temperature was 1.09 °C higher than between 1850 and 1900 [2]. This increasing temperature will likely generate hot climate conditions, especially in tropical areas. Between 2030 and 2050, global warming will probably reach another 1.5 °C if it increases continuously at the current rate [3]. As global warming increases, it is anticipated that every country will experience more simultaneous and complicated alterations in the climatic effect drivers. Climate change can have adverse effects on the animal health, which can result in far-reaching consequences for human health, livelihoods, productivity, and greenhouse gas emissions. To cope with climate change, the genes of animals and plants are influenced by phenotypic plasticity, which enables a gene to produce different phenotypes based on environmental, biotic, and abiotic conditions [4].

Among the domesticated birds, poultry is the most important, which we raise for meat, eggs, or feathers. Poultry meat and egg consumption will be doubled by 2050 [5]. In recent years, poultry genetics has dramatically improved to maintain this vast demand–supply chain. The chicken weighed 905 g in 56 d in 1957; in 2005, it weighed 4,202 g in 56 d, around a 400% hike in growth rate. It also saw a 50% reduction in the feed conversion ratio during this time [6]. To increase this weight, the broiler needs a higher metabolic rate. Chickens need to feed rapidly to maintain a higher metabolic rate. This elevated feeding frequency can raise core body temperature [7]. In addition, higher stocking density, no sweat glands, and exposure to high ambient temperatures harm poultry, making birds vulnerable to heat stress (HS). It can impact physiological pathways through genes that encode leptin, thyroid hormone receptor, insulin growth factor-1, and growth hormone receptor. It can also cause immunological and behavioral changes, which negatively impact the production performance of poultry [4]. HS increases the feed conversion ratio and reduces body weight (BW), feed intake, overall growth performance, and carcass quality, which is harmful to both the poultry industry and consumer health [8]. This decreased growth performance leads to a significant economic loss in the chicken industry [9–12]. In early 2000, heat stress caused economic losses of $128–160 million in the US poultry industry and $1.69–2.20 billion in the US animal industry [13].

Several strategies have been used to mitigate this heat stress effect in poultry. For example, different feeding strategies and nutritional manipulation types have been tested. Supplementation of vitamins, minerals, electrolytes, phytochemicals, and osmolytes is notable. Also, the genetic approach and different managerial strategies like ventilation, housing, reducing stocking density, surface wetting techniques, and litter management are significantly critical [9, 11, 14–17]. One effective way to mitigate heat stress is to breed animals with high heat tolerance. This can be done by selecting individuals from highly productive populations that are already heat-tolerant, rather than relying on crossbreeding or introgression with local breeds [18]. However, thermal manipulation (TM) is another very effective strategy that can mitigate the consequences of heat stress on the body.

TM of chicken embryos at an early developmental stage is acknowledged as a unique management strategy that aids quickly developing broiler chicks to adapt to unfavorable environmental conditions [19]. A program of early heat exposure during embryonic development has been proposed to promote avian thermal toleration and well-being without affecting post-hatch development [20]. As an example, during embryogenesis, the TM treatment consists of increasing the egg incubation temperature to 39.5 °C for 12 h/d, as contrasted to 37.8 °C under conventional settings, during the development of the hypothalamic-pituitary-thyroid axis (HPT-axis) between embryonic day (ED) 7 and ED 16; hatching is around ED 21 [20, 21]. Heat stress at slaughter age (5–6 weeks) modifies physiological parameters in broilers, reducing internal temperature and increasing heat tolerance, with little impact on chick performance.

Several models are suggested for improving the thermotolerance of birds, such as acclimation [22], regulation of the production of heat shock proteins [23], thermal conditioning at an early age [24], and TM at different stages of embryogenesis in the broiler. According to studies, exposing embryos to low or high temperatures during development can enhance their ability to adapt to cold or hot environmental conditions later in life [22–30].

As there is scientific evidence about the beneficial effects of TM in embryonic stages, it is high time to review the results of TM in different stages of broiler life for researchers and the poultry industry. Therefore, the overarching goal of this review paper is (1) to summarize the available literature on TM and (2) its beneficial impact on poultry health and production performance.

## Thermoregulation in poultry

In avian species, the thermoregulatory system is controlled by both neural processes and hormonal interaction. To understand the TM technique, it is essential to understand the thermoregulatory mechanism in poultry clearly. The HPT-axis and the hypothalamic-pituitary-adrenal axis (HPA-axis) must be regarded in this crucial event during the prenatal and perinatal life of birds [31]. The hypothalamus is primarily responsible for coordinating thermoregulation in birds [32]. It has been determined that the preoptic anterior hypothalamic (PO/AH) region of the brain is highly responsive to variations in its local tissue temperature. These cells are called ‘thermoresponsive neurons’ or ‘thermoreceptors’ and are classified as insensitive, warm-sensitive, and cold-sensitive [33]. The preoptic region, located inside and close to the rostral hypothalamus, serves as a coordinating center and exerts major impacts on each lower effector area. Some neurons in the preoptic region are sensitive to minute variations in hypothalamic core temperature. In addition to receiving abundant somatosensory input from cutaneous and spinal thermoreceptors, thermosensitive preoptic neurons also receive a substantial amount of somatosensory input. In this manner, preoptic neurons compare and integrate central and peripheral thermal information. Due to this sensory integration and its control over lower effector areas, the preoptic region triggers thermoregulatory responses that are optimal for internal and external thermal conditions [33].

Thermoreceptor stimulation is triggered by PO/AH. Then, PO/AH activates the hypothalamic paraventricular nucleus (PVN), which increases the synthesis and secretion of the thyrotropin-releasing hormone (TRH). The avian thyroid gland is controlled primarily by the HPT-axis. Thyroid hormones (THs, triiodothyronine, T_3_, thyroxine, and T_4_) are the most crucial hormones regulating thermogenesis; this relationship was established in the late 1950s [34]. The avian hypothalamus secretes TRH and somatotropin release-inhibiting hormone (SRIH), which stimulate and inhibit the pituitary gland, respectively [31]. TRH stimulates the anterior pituitary thyrotrophs to release thyroid stimulating hormone (TSH) that interacts with thyroid follicular cell membrane receptors. This increases the production and secretion of thyroid hormone (primarily T_4_). T_4_ is the precursor form of the biologically active form T_3_. Deiodinase enzymes (DIO1 and DIO2) convert T_4_ to T_3_ within tissues. A negative feedback mechanism of T_3_ modulates TSH secretion by the pituitary in addition to hypothalamic TRH [35]. In contrast, cold exposure can promote the conversion of T_4_ to T_3_ (and less to inactive reverse T_3_) by deiodinase enzymes (DIO3) in tissues, particularly the liver, which increases circulating T_3_ concentration. T_3_ attaches to nuclear and mitochondrial receptors in tissues and modulates gene expression to control metabolic rates and respiration [36].

**Fig. 1:**
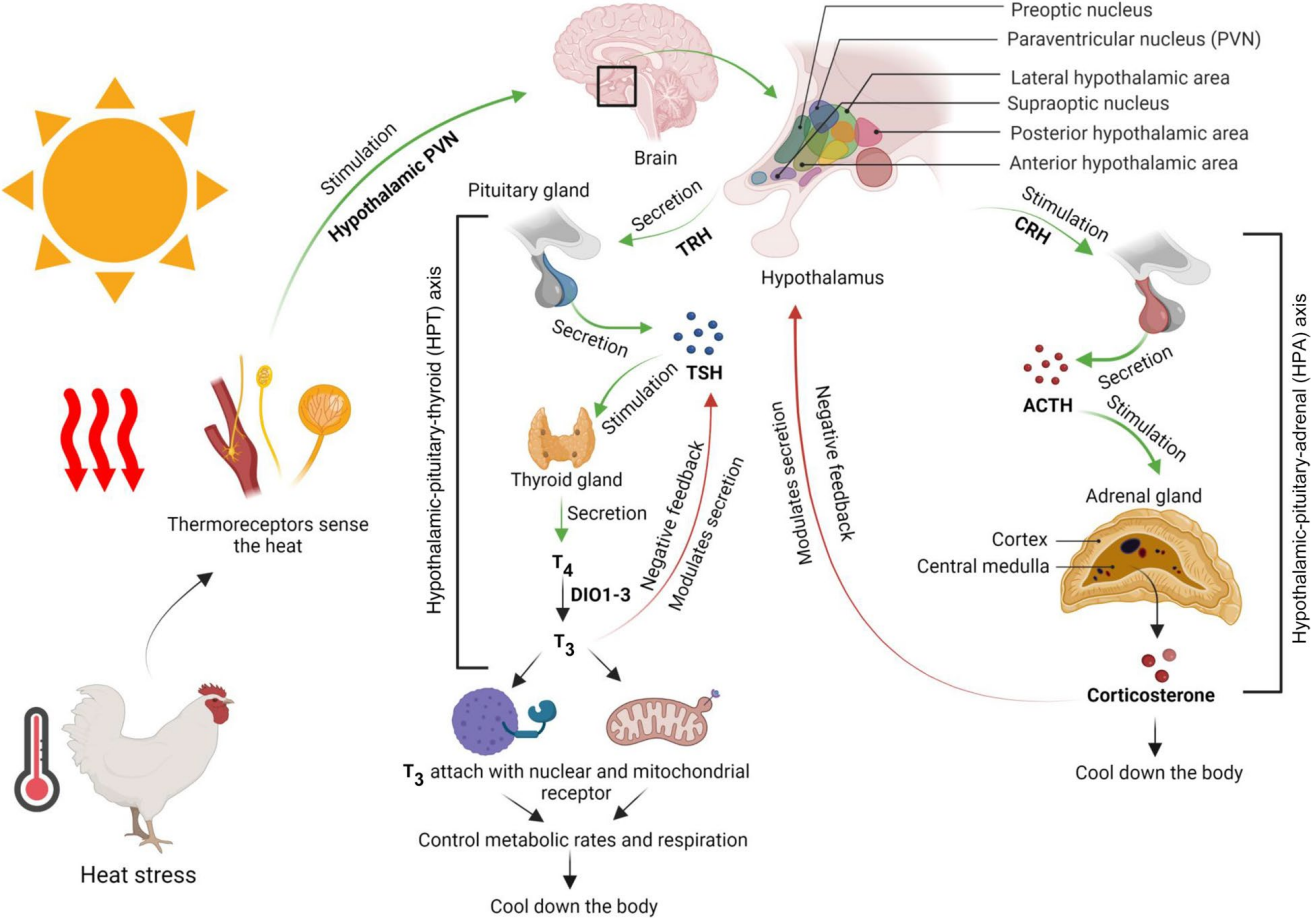
Mechanism of thermoregulation in poultry (Created by Biorender.com)

In addition, the hypothalamus generates corticotropin-releasing hormone (CRH) as a part of stress response. The anterior pituitary produces adrenocorticotropic hormone, which the hypothalamus stimulates. The release of adrenocorticotropic hormone stimulates the adrenal cortex, resulting in a spike in corticosterone, a hormone that exerts negative feedback on both the hypothalamus and the pituitary gland [37]. Through the specific CRH-receptor 2, CRH also stimulates the anterior pituitary to produce TSH in 19-day-old chicken embryos, indicating that the HPT and HPA-axes interact strongly throughout prenatal phases [38] (Fig. 1).

## Heat stress response in poultry

**Fig. 2:**
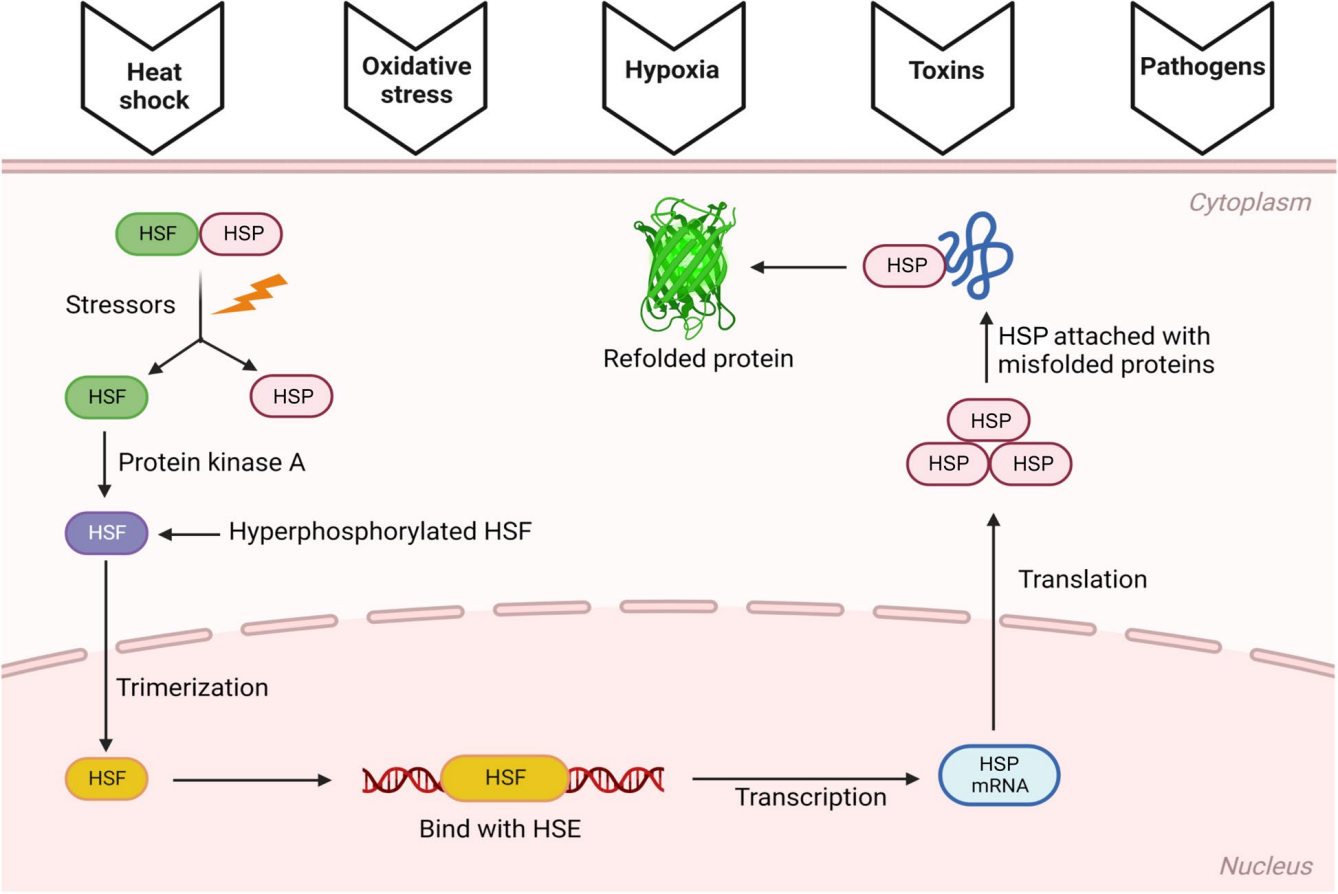
Mechanism of heat shock factor (HSF) in the body (Created by Biorender.com)

The heat shock response (HSR) is one of the primary adaptive stress responses of cell homeostasis restoration following proteotoxic stress, such as heat shock, cold, oxidative stress, hypoxia, toxins, compounds, and pathogens [39]. The cellular stress response is dependent on the structure and function of proteins. One of the most prevalent characteristics of the cellular stress response is the production of heat shock proteins (HSPs) in response to stressors. HSPs can significantly alter the physiological stress response and promote tolerance [40, 41]. HSPs are a diverse class of molecular chaperones with various molecular weights and biological roles. Small HSPs (molecular weight 540 kDa) and the HSP60, HSP70, HSP90, and HSP100 protein families are typical divisions of HSPs. These families all operate as chaperones, facilitating the folding, unfolding, and refolding of newly formed or stressed-out proteins to stop erroneous folding and additional deterioration and damage [42]. At the transcriptional level, heat shock response is primarily regulated by four heat shock transcription factors (HSFs), including HSF1, HSF2, HSF3, and HSF4 [43]. The HSP–HSF complex dissociates to an active state in the cytosol during stress. Once activated, they get hyperphosphorylated by protein kinases, translocated to the nucleus, and trimerized [44]. The HSF–HSF-phosphorylated trimer complex reaches the nucleus and activates the HSP gene by binding to the heat shock element found in the promoter of the HSP gene. HSP mRNA is then translated and released from the nucleus to the cytosol, where new HSP is synthesized. This results in the proliferation of HSPs within the cell, which assists in refolding the damaged proteins [45] (Fig. 2).

Heat shock proteins are one of the most critical indicators of heat stress in the body. A list of essential heat shock proteins and genes is shown in Table 1.

**Table 1:** Functions of heat shock proteins and genes involved in heat stress in chicken

Genes involved	Molecular function	Biological process	References
*HSP25*	Inhibit actin polymerization, lower the cholinergic moto neurons	Protective function against heat shock, actin filaments rearrange themselves rapidly in response to heat stress	[46]
*HSP27*	Acts as an anti-apoptotic agent to remodel actin cytoskeleton and promotes actin polymerization	Cellular ability to respond to heat, rising intracellular glutathione, and lowering the intracellular iron level	[47]
*HSP47*	Binding protease, activation of endopeptidase inhibitor	Proteolysis, the metabolic process of cellular protein, negatively regulates endopeptidase activity	[48]
*HSP70* *HSPA2*	Binding ATP codified heat shock protein, binding unfolded protein, and processing ATPase	Vesicle-influenced transportation, folding of the chaperone-mediated protein, cellular response for the unfolded protein	[48]
*HSP100*	Disassemble higher-order protein structures, inhibition of leukotoxin expression	Increase tolerance to high temperature, promotion of proteolysis	[49]
*HSF1*	Transcription factor activity for binding DNA, DNA binding by RNA polymerase II at the proximal promoter sequence	The capacity of cells to respond to heat, transcription from the RNA polymerase II activator in reaction to stress, transcription by RNA polymerase, and RNA polymerase II-mediated positive control of transcription	[48]
*HSF2*	Transcription regulation of heat shock response	Modify the expression of HSP70 through interaction with its chromatin	[50]
*HSF3*	Transcription factor activity for binding DNA, RNA polymerase II proximal promoter sequencer-specific DNA binding	The capacity of cells to respond to heat, transcription from the RNA polymerase II promoter in reaction to stress, transcription by RNA polymerase, and RNA polymerase II-mediated positive control of transcription	[48]

## Mechanism of TM

**Fig. 3:**
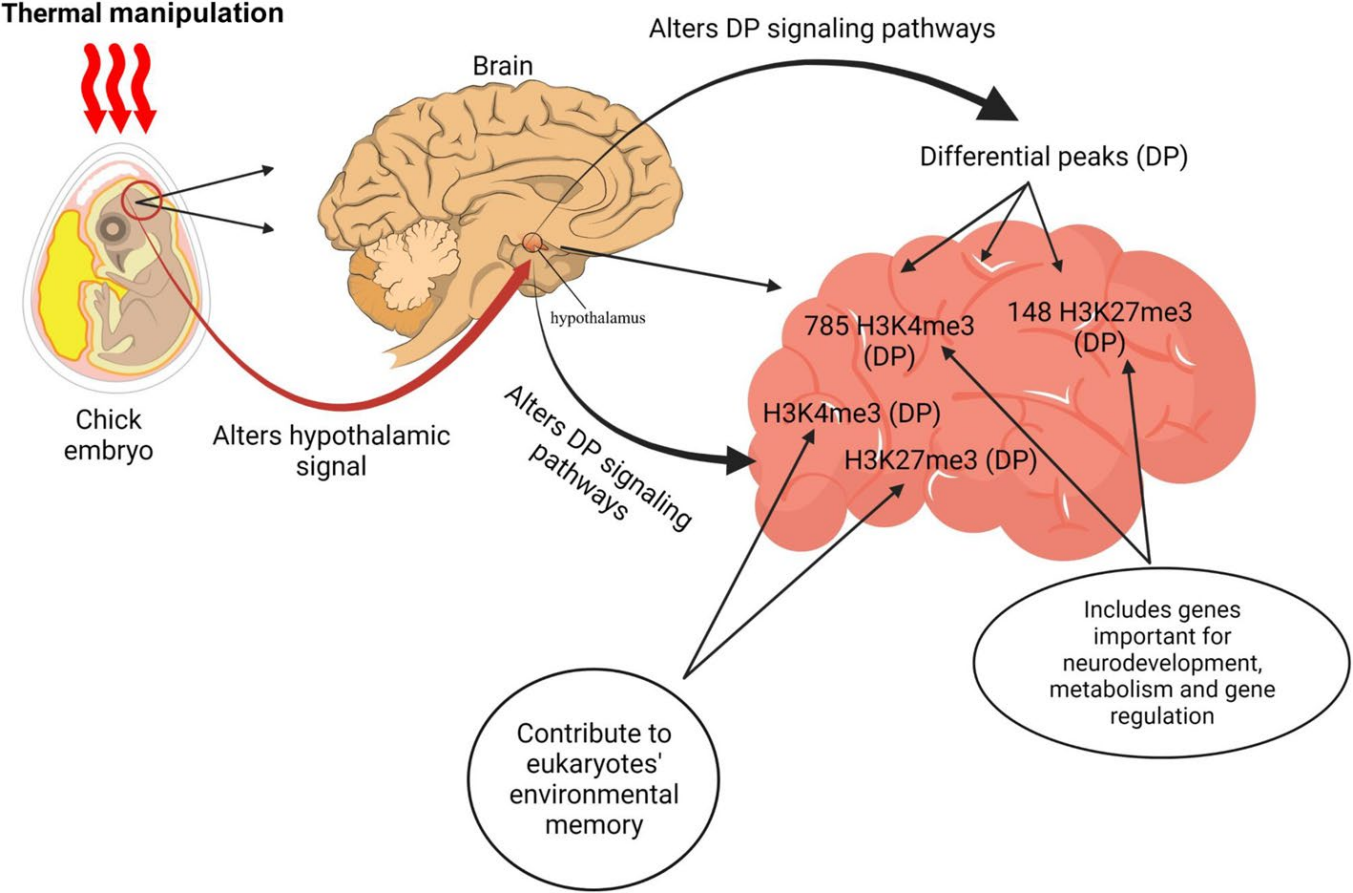
Mechanism of thermal manipulation (TM) in poultry (Created by Biorender.com)

The distinctive nature of the primary germ cells in chickens presents unique possibilities for modifying the epigenome of the developing embryo by manipulating its environment during the early stages. Embryonic TM in poultry primarily functions by triggering epigenetic alterations [51]. Epigenetic modifications refer to alterations in the phenotype that are not associated with changes in the DNA sequence [52]. These alterations are crucial for governing cellular differentiation and organism development. Epigenetics controls the utilization of an animal’s genetic potential in this scenario. The primary epigenetic mechanisms encompass microRNA activity, DNA methylation, and histone alteration. TM predominantly influences epigenetic modifications in broiler embryos via DNA methylation and histone modification [53]. DNA methylation is the most extensively researched epigenetic process. This reliable epigenetic indicator can be passed down through multiple rounds of cell division [54]. DNA methylation primarily takes place at the dinucleotide site of promoter CpG. When DNA methyltransferase is present, cytosine’s 5′ carbon site forms a covalent bond with a methyl group, forming 5-methylcytosine (5mc). CpG methylation in the distal region of the promoter hinders protein binding at these methylated sites, thereby impeding gene transcription. DNA demethylation is reliant on the activity of the ten-eleven translocation enzyme family, which can transform 5-methylcytosine (5mc) into 5-hydroxymethylcytosine (5hmc). This conversion is a crucial step in the demethylation process [55]. Histone acetylation is a process that alters the structure of chromatin and plays a role in DNA replication and repair, gene transcription, and the suppression of heterochromatin. Histone deacetylase is an enzymatic catalyst that eliminates the acetyl moiety from histone proteins bound to DNA. This hinders the accessibility of DNA to transcription factors [56]. Two post-translational modifications of histones, H3K4me3 and H3K27me3, are present in brain and muscle tissues and contribute to the environmental memory of eukaryotes, H3K4me3 and H3K27me3 are found in the brain and muscle tissues and contribute to eukaryotes’ environmental memory. The hypothalamus has 785 H3K4me3 and 148 H3K27me3 differential peaks (DP), including genes important in neurodevelopment, metabolism, and gene regulation. In TM animals, most DP had decreased signals for markings and tissues. H3K4me3 DP was detected in the hypothalamus mostly at transcription start sites, the first exon, and the first intron of genes. Only DP outside of genes was found to be underrepresented, suggesting that H3K4me3 alterations occurred preferentially within genes inside the hypothalamus of TM animals. However, H3K27me3 hypothalamic DP was equally scattered across the body of genes, most likely due to the larger peak sizes. The response of chicken embryos to pre-hatch TM is partially regulated by hypothalamic epigenetic alterations, which may contribute to post-hatch thermal acclimatization [57] (Fig. 3).

The process of heat acclimation may be influenced by epigenetic regulation of gene expression. This mechanism may impact the expression of specific crucial genes in response to heat, thereby regulating phenotypic changes and facilitating the development of heat tolerance. However, the exact molecular mechanism of TM is still incomplete. There is very little information available. Additional research is required to explore the potential connections between TM and epigenetic modification.

## Application of TM

The classic Hamburger and Hamilton Staging Table is extensively used to classify the 46 stages of chick embryo development. Different prominent morphological changes and developmental characteristics were used to classify distinct phases of development. The development of the primitive streak characterizes the initial phases (1 to 6 stages from 0 to 24 h). The middle phases (7 to 14 stages from 24 to 53 h) are primarily defined by the number of somites and associated characteristics. The late developmental stages (15 to 45 from 53 h till hatch) are distinguished by various morphological traits and categorized by standard measurable characteristics [58].

The best timeline to implement the TM strategy is the mid or late embryonic stage. At ED 15 and 20, the embryo can respond to variations in incubation temperature. In this period, thermoregulation occurs as a negative feedback control system [59]. The early embryonic stage might not be practical because, at that stage, the hypothalamus has not yet been developed to acquire the epigenetic change during the TM. Therefore, it is crucial to know all the stages of embryogenesis for a successful thermal manipulation strategy (Table 2).

**Table 2:** Important changes during embryogenesis in chicken

**Days**	**Development Stages**	References
1 and 2	The heart is formed and starts beating. Central nervous system (CNS) formation began	[58, 60–62]
3	The limbs burgeon from the wings, and the auditory cleft is established, indicating the development of the skeletal muscular system. Embryo movement represents the interaction of skeletal, muscular, and nervous systems	
4	Joint cavity, cartilage, and bone formation	
5 and 6	The emergence of reproductive organs, sexual differentiation, and initiation of voluntary movements starts	
7	The heart is entirely enclosed within the thoracic cavity, and feather filaments are beginning to form. Thyroid and adrenal glands start to function	
8 and 9	Starts formation of digits and third toe. Formation of preoptic-vestibule. Heat production from embryos increases	
10	The beak hardens, and the toes are entirely developed	
11 and 12	Formation of the auditory system	
13 and 14	There is not much morphological change. Endogenous oscillations around peripheral synaptogenesis initiate the ontogeny of hearing	
15 and 16	Embryos respond to external sounds and a wide range of frequencies	
17	The renal system starts producing urate	
18	The embryo rotates its body to the correct position. Neck and head folded beneath the air space membrane	
19	Hatching behavior becomes more prominent, and internal pipping starts	
20 and 21	Beginning of pulmonary respiration. External pipping and finally hatching	

**Fig. 4:**
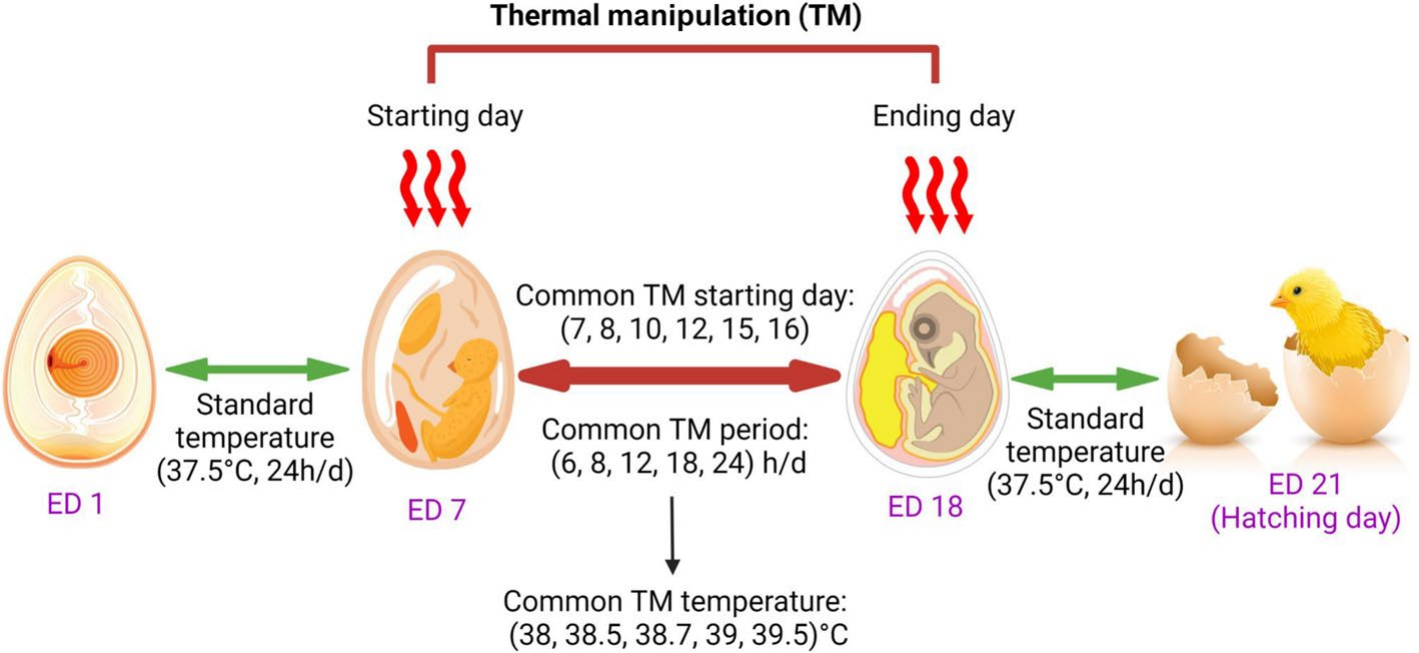
Application of thermal manipulation (TM) strategy in broiler chicken (Created by Biorender.com)

Researchers have tried different TM strategies for an extended period. Some common TM temperatures and timelines are shown in Fig. 4.

## Effect of TM on poultry health and production

Significant physiological changes that take place in the TM birds are:

### Effects of TM on hatchability

Fertility and hatchability are two main factors that significantly impact the availability of day-old chicks. Fertility indicates the percentage of fertile eggs out of all the eggs after candling, whereas hatchability refers to the rate at which fertile eggs hatch after incubation. Strain, nutrition, health, egg size, weight and quality, and egg storage conditions impact hatchability in broiler chicken production—also, too high an incubation temperature results in lower hatchability [63]. However, the impact of an increase in incubation temperature on hatchability is contingent on the duration and magnitude of the temperature increase. Moreover, the stage of embryogenesis at which the temperature change occurs. Various experiments using a short-term temperature rise during multiple stages of incubation have shown conflicting findings regarding hatchability and hatching time. Continuous TM (24 h/d) caused reduced hatchability (only 63%) and delayed hatching with lower BW because extended and constant exposure to TM resulted in inadequate yolk sac absorption. However, intermitted TM (12 h) did not affect chick quality or hatchability (83%) [64]. TM at 39.5 °C and 65% RH for 12 h from ED 7 to ED 16 significantly increases embryo mortality and decreases hatchability compared to 37.5 °C and 56% RH [65]. The elevated eggshell temperature observed in the TM group following ED 11 indicates that the embryo’s growth was constrained during the 12 h exposure to TM. The measurement of eggshell temperature is utilized to measure the temperature encountered by the embryo. This measurement is directly correlated with the metabolic activity of the embryo during the latter portion of the incubation period [66]. This might be the reason for lower hatchability and higher embryo mortality. In a study, (38.2 to 38.4 °C at ED 18–21 for 24 h/d) and (38.2 to 38.4 ℃ at ED18–21 for 2 h/d) incubation period has shown no significant difference in hatchability (around 95%). However, transient warmth simulation improved hatchability by more than 1.5% [67]. However, hatchability was reduced when hatching eggs were exposed to high temperatures for an extended time during the initial phases of incubation [68]. During the final stages of incubation in poultry embryos, the peripheral and central nervous thermoregulatory mechanisms and other body functions are highly developed [69]. This may explain the differences in hatchability observed during the first and final incubation phases. Applying heat treatments before (30.2 °C for 12 h/d) or during the sex determination phase of incubation (38.1 °C between ED 0 and ED 5) increased the hatchability [70]. TM did not affect the hatchability percentage of total and viable set eggs, the percentage of normal birds, chick quality, and body temperature (°C) at hatch. The experiments were conducted during winter and summer, with Cobb 500 fertile eggs stored for 4 and 9 d, respectively. The egg storage time might be the reason for the higher hatchability. In our study, TM at 38.5 °C and 65% RH for 12 h/d from ED 10 to 18 significantly increased hatchability (94.5%) and reduces hatch time (by 6 h) [71]. The potential reason could be attributed to the higher metabolism in TM embryos, resulting in reduced embryo mortality and increased hatchability.

Hatchability is a crucial parameter for the industry. The high hatching rate directly relates to the profit margin and the consumers’ price determination. The industry will not adopt the TM technique if it cannot increase the hatchability. TM temperatures above 40 °C and below 34 °C can sacrifice hatchability. Too high temperature (above 40 °C) and too low temperature (below 34 °C) increase stress in developing embryos, hinder embryonic metabolism, and cause embryonic mortality. TM period is also crucial in this case. Long-term TM starting from the early embryonic stage (ED 1–9) lowers the hatching percentage, and short-term TM does not affect it. So, the TM should be applied at the mid to late embryonic stage (ED 10–20), the application timeline should be short (6–16 h), and the temperature should be moderate (38–40 °C) to achieve a desirable hatching rate without negatively impacting the hatchability.

### Effects of TM on sex ratio

The ratio of females to the number of males in each population is called the sex ratio. The sex ratio affects wild population growth rates and evolutionary trajectories [72]. The most prevalent evolutionary stable strategy is the generation of males and females in a 1:1 ratio, forced by frequency-dependent natural selection because of competition for mates among individuals of the same sex [72]. TM has been shown to affect broilers’ sex ratio; it benefits the industry. Male embryos usually hatch later than female embryos; TM accelerates embryo development. So, thermal treatment enhanced male percentages and male/female ratios more than the control group during ED 19–21 [73]. The incubation temperature affects the sex ratio in Australian brush turkeys (*Alectura lathami*), a species of mound-building megapode. The mean incubation temperature in natural mounds is approximately 34 °C. However, at temperatures of 31 and 36 °C, the sex ratios were modified. A higher proportion of males hatched at a temperature of 31 °C, while a higher proportion of females hatched at 36 °C. At 34 °C, the ratio of males to females was nearly equal [74]. The skewed sex ratio can be attributed to variations in embryonic mortality. There were two experiment setups in one study; if the temperature is increased by 0.5 °C, it increases the male chicks’ hatchability by 3.5% in the first experiment and 2.2% in another investigation [75]. However, eggs incubated in slightly lower (36.7 °C), standard (37.5 °C), and higher (38.3 °C) temperatures had similar percentages of male chicks. Male hatchability is 49.8% in the cold treatment, 51.4% at standard temperature, and 49.5% in the heat treatment [76]. TM at 38.2 °C at ED 18–21 for 4 h/d resulted in a significantly higher proportion of male chicks hatched than the control group [77]. However, TM at 36.2 °C at ED15–21 for 2 h/d showed an opposite result, with more female chicks than males [78]. The discrepancies of both studies need further investigation.

The process of determining the sex of the undifferentiated reproductive organ in the chick is believed to take place within the initial week of incubation. Changes in the concentration of hormones (possibly lower corticosterone level) connected to embryo metabolism and development may impact the sex ratio. It may have improved the hatchability of male chicks, especially in males around piping time, and resulted in a higher male proportion [75, 77]. One of the vital aspects of TM is that it has a considerable impact on the sex ratio in broiler birds. High and cold TM temperature has some influence on the sex ratio. However, there is not much evidence of TM changing the sex ratio in broiler chickens. So, the effect of TM on the sex ratio is still unknown. More studies should focus on this aspect of TM before we can conclude anything about it.

### Effects of TM on behaviour and welfare

Artificial incubation of chicken eggs is a meticulously managed process that requires monitoring and regulating various parameters. The commonly utilized incubation temperature of 37.6 °C has been established commercially. Recognized for its ability to optimize hatchability, it is commonly utilized as the default temperature setting in modern commercial incubators. However, many studies have been done on the effects of higher and lower incubation temperatures on the chicken’s heat/cold resilience and/or production traits later in life [79, 80]. Before implementing the technique, it is crucial to ensure that the manipulation causes no harmful effects. The animal’s performance, behavior, and welfare must remain unharmed.

In a study, the non-optimal Incubation Treatment (IT) group (TM at 36.6 °C for 24 h/d from ED 13 to 18) and the natural treatment (NT) group (TM at 30 °C for 30 min/d from ED 13 to 18) were tested for post-hatch fear and social behavior [81]. Their quantification involved six scientifically validated behavioral tests: three fear tests (novel object on d 1 and 8, tonic immobility on d 40, inversion on d 42), two social tests (isolation on d 19 and social reinstatement on d 25), and one social/fear test (emergence on d 18 and 20). In the emergence test, a treatment effect was observed on the emergence latency, with Control chicks emerging more frequently and faster than IT chicks. The emergence test measures the time it takes for a single chick to emerge from a T-shaped box. Since no disparities in IT were observed in other social assessments, the findings indicate that IT chicks may exhibit greater reluctance towards unfamiliar surroundings. No distinctions were found between NT and Control or NT and IT. An interaction was observed between treatment and sex during the isolation test, where a single chick was isolated visually and audibly for 3 min, and the number of vocalizations was recorded. Female individuals exhibited a higher frequency of vocalizations than males in the IT and C groups but not in the NT group. Male chicks from the NT may be more sensitive to social separation due to the increased effect of early stress caused by severe environmental temperature fluctuations. This stress is likely to have a greater impact on the anxiety behavior of males compared to females later in life. Two different TM temperatures, T1 (38.6 °C for 8 h/d between ED 15–20) and T2 (39.6 °C for 8 h/d between ED 15–20), were also tested for seven behavioral experiments [82]. The experiment includes a human approach test, an emergence test, a social reinstatement test, an isolation test, a novel arena test, and a novel object test. These tests were selected and replicated two to three times. The study findings indicated that the interaction between gender and treatment impacted social behaviors. Males exhibit a decreased propensity for social reinstatement during standard incubation; however, both treatments resulted in behavioral changes. In isolation, males from group T1 expressed themselves significantly higher than females (73 ± 4 calls vs. 38 ± 2 calls). Furthermore, during a social reinstatement test, males from group T2 demonstrated significantly greater social motivation than those from groups Control (T2: 121 ± 20 s vs. Control: 66 ± 25 s) and T1 (57 ± 27 s). An age effect was ultimately identified, whereby older birds exhibited reduced fear in response to novel objects and environments but heightened fear in response to an approaching human. They appear to be more socially motivated to interact with conspecifics. Habituation, alterations in sensory and/or cognitive abilities, variation between repetitions, and potential decreased mobility in old age may all impact the behavioral response over time, casting doubt on the reliability of these age-related findings.

White Leghorn laying hens exposed to TM at 27.2 °C for 1 h/d between ED 12–19 exhibited higher responses to new situations than the control chicks [83]. This was demonstrated by longer latencies in initiating movement in the open-field test and approaching novel food, indicating higher levels of fearfulness. Social motivation and discrimination did not differ significantly between the treatment and control groups. Nevertheless, this outcome was unforeseen since both factors are crucial for the social adaptation of gregarious animals. However, TM at 40 °C for 4 h/d between ED 14–18 noticed no significant variations in the behavior of laying chickens, especially when experiencing heat stress in post-hatch [28]. This indicates that laying hens probably have a more efficient thermotolerance due to their slower metabolism and growth rate. Another study examined three incubation temperatures (TM at 35.0, 35.8, and 37.0 °C for 24 h/d) that occurred naturally but were not optimal for wood ducks (*Aix sponsa*) [84]. The results indicated that the animals exhibited more proactive behavior at the lowest temperature, unlike the other two. This effect was particularly pronounced in younger ducklings and males. This demonstrated behavior may be critical for their survival.

Although an elevation in temperature during incubation may lead to enhanced heat tolerance in adulthood, it also has the potential to impair social behavior. The impact of TM on poultry behavior and welfare after hatching has received scant attention. Therefore, it is unwise to draw any conclusions regarding the welfare and behavior of TM birds in the post-hatch period. The welfare and behavioral patterns of poultry may also have an impact on their heat tolerance, which merits further investigation.

### Effects of TM on growth performance

In broiler production, growth performances refer to the chickens’ measurable and observable traits throughout their growth and development phase. The performance characteristics mainly include feed intake, body weight gain, meat yield, bone growth, and feed conversation ratio (FCR). Supplying the birds with adequate feed and receiving regular growth performance are the keys to successful broiler production in the industry. So, growth performance is one of the most critical parameters for the poultry industry. Thermal conditioning has been presented as a low-cost method of improving broiler performance and thermotolerance; compared to the control, thermal conditioning considerably enhanced body weight gain and feed intake [85].

Final BW is crucial in determining overall growth performance in chickens. BW enhancement in broiler chicken dramatically depends on TM. The perfect synchronization of duration, timing, and temperature plays a vital role in achieving the desired pre-hatch TM. It helps thermal acquisition during weight gain [86]. TM at 39.5 °C and 65% RH for 12 h/d from ED 7 to 16 significantly decreases the hatching BW [87]. It may be attributed to differences in sex, specifically male versus female. It is suggested that males are more capable of compensatory gain than females [20]. TM at 38.5 °C for 6 h/d at ED 16 and 9 h/d at ED 17 significantly improved BW on d 4, 5, and 7. TM at 40 °C for 6 h at ED 16, 9 h at ED 17, and 12 h/d at ED 18 significantly increased BW on d 14, 28, and 42 [25]. Ross 308 broiler incubation between the ED 18 and 20, acute stimulation (2 h/d) of 1 °C above 37.4 °C incubation temperature, resulted in no significant changes in chick quality. Heat manipulation therapy improved the performance of male chicks and significantly lowered the FCR ratio compared to control chicks (37.4 °C) [77]. In quails, all the thermally treated (41 °C at 65% RH for 3 h/d at ED 12–14) chicks had considerably lower hatching weights than the control chicks. However, early TM dramatically increased body weight at d 35 compared to other groups [88]. The TM protocol was applied during the late embryogenesis period in these studies. The late embryonic period is the final stage in the development of the HPT-axis and HPA-axis, which play a crucial role in metabolism, thermoregulation, and response to stress. Also, it is the period of fetal myoblast development and the primary period for the development of satellite muscle cells [89]. These factors contribute to the final muscle mass and its capacity for hypertrophy. TM did not affect BW at 39 °C on d 21 and 42 [90]. The low T_3_ levels observed in the chickens from the high incubation treatment appear to be a thermoregulatory mechanism employed by these chickens to decrease their feed intake and, as a result, reduce metabolic heat production. In rats, a decrease in thyroid hormone levels leads to decreased food intake, body weight, and body temperature [91]. So, it is anticipated that chicks with lower metabolism will have lower feed intake rates and slower growth rates. Broilers incubated at high temperatures between ED 7–10 or ED 10–13 at 38.8 °C had the most significant body weight than the eggs incubated at 37.8 and 36.8 °C, respectively [92]. During incubation, chicks exposed to 1.39 °C above the standard suggested temperature at ED 14–18 had better quality and performance [93]. These studies showed a higher egg yolk weight at hatch. Immediately after hatching, the chicks rely on the remaining yolk for nutrition rather than consuming feed [94], which might have impacted their growth in this study. The broilers exposed to incubation at 38.5 °C for 6 h/d from ED 10 to 18 have lower BW than those at 37.8 ℃. The lower BW could be explained by variations in chicken strains, timing, temperature, or TM conditions’ duration in this study [95]. The BW of both sexes was unaffected by thermal treatment; however, males substantially gained more weight than females. When chicks of each sex or treatment were sorted according to hatching time, the late-hatching females were significantly heavier than the others [86]. Higher TM temperature may decrease final body weight in the chickens and cause embryo death. However, if the higher temperature can synchronize with perfect timing, it can improve body weight in the marketing age. High incubation temperature leads to high eggshell temperature (EST), and high EST (38.9 °C) from d 7 to 21 of incubation decreases the growth performance but increases breast meat yield [96]. This phenomenon could potentially be linked to enhanced muscle cell proliferation and expedited differentiation following prolonged exposure to high EST [97]. Different placements of the incubation tray can increase the incubation temperature. It was reported that eggs were incubated in hatching trays kept in the racks’ bottom, middle, and top thirds at 38.09, 38.24, and 37.99 °C, respectively [98]. The changes in incubation temperature between tray placements may not be sufficient to alter the overall success of post-hatch development. The intrinsic temperature resistance of avian embryos might explain these findings.

In a TM treatment, the incubation temperature was elevated to 39.5 °C, and the RH was increased to 65% for 12 h/d from ED 7 to 16 except for weeks 1, 2 and 4. TM-treated males’ feed intake was lower than that of control males. Except for weeks 1, 2, 4, and 7, females showed a similar pattern in feed consumption. The FCR followed the same statistical pattern as feed consumption in both sexes. Compared to the TM broiler, the total FCR was lower in the control group [99]. Like the high TM during the incubation stage, the group constantly exposed to postnatal cold temperature exhibited a 5% significantly greater feed consumption ratio. On ED 18 and 19, eggs were incubated at 15 °C and 81% RH for 30 min before being transferred to 37.6 °C and 56% RH for the rest of the incubation time. The incubation environment and postnatal ambient temperature had little effect on the feed intake of both sexes. In cyclically cold postnatal environments, cold incubation increased female feed efficiency by 4% relative to the control group [100]. The event was most likely caused by increased heat production to counteract the effects of cold to maintain a normal body temperature (thermoregulation) [101]. During the first two weeks after hatching, the chicks that received high heat treatment had rectal temperatures, which did not affect these chickens’ growth rate and feed intake because they were thermotolerant [90].

Bone formation is another important aspect of growth in broiler chickens. It is well known that embryonic development and growth are accelerated at higher temperature incubation. It is reported that on the 20^th^ days of embryonic development or at hatching, there were no appreciable variations in femur lengths between treatment groups. During ED 20, the facial lengths showed comparable results. The lengths of the tibia and metatarsus were significantly longer in the thermal manipulation groups than in the control group [102]. Treatments included incubating eggs at 37.8 °C and 55% RH continuously (control), 36.9 °C and 60% RH for 6 h/d from ED 0 to 8, 36.9 °C and 60% RH for 6 h/d from ED 10 to 18, 41 °C and 65% RH for 3 h/d from ED 8 to 10, and 41 °C and 65% RH for 3 h/d from ED 10 to 18 [102]. However, another study found that high temperature (39 °C for 24 h/d from ED 13 to 21) significantly decreased the body weight, mineral concentrations in both bones, femoral fracture strength, and tibiotarsal stiffness [103]. The bone growth process involves intramembranous and endochondral mechanisms [104]. The reduction in both the length and diameter of the femoral and tibiotarsal diaphysis in birds reared under high temperature indicates that elevated rearing temperature impacts the growth processes of leg bones, specifically the intramembranous and endochondral processes.

So, to improve growth performance, we need to improve the FCR. TM emerges as a promising strategy to increase growth performances post-hatch. As TM improves the thermotolerance in broiler chicken, it does not sacrifice the feed intake. However, only TM cannot enhance growth performance; post-hatch management strategies are also essential. If the feeding time, formulation, and stocking density are correct, TM birds can perform satisfactorily.

### Effects of TM on muscle growth and development

Muscle growth drives meat production, including protein accumulation, myofiber hypertrophy, and muscle-cell multiplication [105]. Muscle progenitors undergo myogenic determination during embryogenesis, resulting in myoblasts proliferating, later differentiating, and merging into multinucleate fibres [106]. On ED 5, chick embryonic myoblasts are the most numerous, but fetal myoblasts are most prevalent between ED 8–12 [89]. Satellite cells (mature myoblasts) are initially recognized in chicks between ED 13–16 when the basal layer surrounding the myofibers matures [107]. As early as ED 8, the influence of the chicks’ prolonged constant subjection to TM was evident in the myofiber radius of the pectoralis muscle**.** The percentage of myofibers with larger diameters was more significant in the control group than in the intervention group (25% vs. 15% within the diameter span of 7 to 13.5 m). By the end of week one post-hatch, chronic heat stress produced fat accumulation in satellite cells arising from the pectoralis muscle, which continued to rise in week 2. The fat deposition was detected in interstitial sections of muscle, probably because of induced adipocyte proliferation or fibroblasts converting to the audiogenic lineage. Additionally, fat deposition was seen in the TM group’s myofibers [108]. The proportion of myofiber diameters in commercial broilers exposed to the TM was comparable to that observed under control conditions, except for a slightly reduced frequency of myofibers with larger diameters (75 to 95 µm) [109].

TM for 12 h/d from ED 7 to 16 at 39.5 °C increases pectoralis minor but showed no significant changes in pectoralis major [110]. However, a significant improvement of breast muscle was observed in shorter TM duration (3 h/d from ED 16 to 18) on 43 d broilers [29]. Another study found that, early heat treatment (EA) (On ED 8, 9, and 10 of embryogenesis, 39.5 °C and 65% RH were administered for 3 h/d), late heat treatment (LA) (On ED 16, 17, and 18 of embryogenesis, 39.5 °C and 65% RH were administered for 3 h/d) and EA-LA treatment (39.5 °C and 65% RH at ED 8 to 10 and ED 16 to 18) the pH of the pectoralis major muscle of the breast was not affected by the thermal challenge but by the incubation treatment. Also, LA broilers had a higher breast meat yield than other groups [29]. The most considerable effect on muscle development occurs when late-term embryos are treated with TM [111]. Between ED 16–18, a daily rise of 1.7 °C above standard settings for 3 or 6 h/d enhanced muscle fiber growth at d 13 and 35 of age [97]. *PAX7* levels were higher in muscle cells and tissue derived from the TM groups than in the control group. It is known that in adult satellite cells, *PAX7* is expressed [97]. It has been reported that this marker indicates the ability of satellite cells to proliferate in turkey poults and broilers [105]. This might enhance the formation of a larger reservoir of myogenic progeny cells, primarily due to the presence of satellite cells. Short-term TM (ED 0–5) improved the relative breast muscle weight of female 35 d broilers but had no impact on male broilers [70]. Similarly, increasing the pre-hatch temperature between ED 4–7 from 37.5 to 38.5 °C enhanced gastrocnemius muscle growth, the number of fibers and nuclei, and the nuclei:fiber ratio in layers. However, the identical TM in broilers resulted in a decreased gastrocnemius cross-sectional area with minimal fibers and nuclei but no change in the nuclei proportion of fibers [112]. This difference in layer and broiler can be explained by the different phenotypic changes that are accompanied by distinct alterations in the expression levels of genes involved in the development and growth of muscles. Further investigation is needed to explore potential interactive mechanisms in the upstream genomic and epigenomic domains. TM applied on ED 16–18 for 3 h/d at 39.5 °C enhanced the comparative weight of pectoralis muscle in female birds 42 d old but had no impact on male birds [29]. One possible explanation is that a rise in temperature from ED 16 to 18 during the proliferation phase of fetal myoblasts [89] may impact the processes of cell proliferation and differentiation, consequently affecting the overall number of fibers in the breast muscle.

TM during ED 10–20 stimulates myogenesis-related gene expression in duck skeletal muscle more than TM during ED 20–27. Embryonic duck leg muscle development is more sensitive to TM than breast muscle. TM may influence the enhancer methylation state of duck *AKIRIN2*, influencing embryonic myogenesis [113]. It is reported that (TM at 38.5 °C for 18 h/d on ED 12–18) and (TM at 39 °C for 18 h/d on ED 12–18) presented the ideal conditions for its growth, increasing the BW. This increase in BW is related to both immediate and long-lasting effects of TM on muscle gene expression. It upregulated *IGF-1,* growth hormone (*GH*), and muscle marker genetics (*MyoD*, *Myogenin*, *PAX7*, and *PCNA*). *MyoD* and *Myogenin* help in cell proliferation [114]. Beyond their role in growth, *GH* and *IGF1* have significant metabolic consequences. They work together to control the metabolism of fat, protein, and glucose because *GH* increases the creation of *IGF1* in various tissues [115]. Also, *PCNA* coordinates many proteins in various DNA-related processes. Several enzymes exhibit a rise in catalytic efficiency due to their interaction with *PCNA* [116]. TM at 38.5 and 39 °C at ED 12–18 significantly upregulated muscle growth factor genes that have long-term effects on increasing muscle development and development [114]. *PAX7* mRNA expression was found to be higher in the heat-treated birds. During embryonic development, the transcription factor *PAX7* is essential for creating and differentiating skeletal muscle precursor cells. It also functions as an anti-apoptotic factor and regulates the expression of other myogenic regulators. Additionally, it contributes to the growth and upkeep of the population of satellite cells. Most significantly, *PAX7* mediates the molecular transition from the embryonic to fetal myoblast development program [117].

In TM (ED 7–11 at 39 °C for 18 h), the histological evaluation of the longitudinal sections thigh muscle fibers radius, pectoral muscle perimysium thickness, and thigh muscle perimysium girth are significantly greater than the control group because TM treatments on breast muscle yield during embryogenesis were beneficial [118]. In addition, satellite cells are skeletal muscle precursors mediating post-hatch muscle development. The temperature impacts their physiological activities. At 50 d of age, Ross 708 broilers show the highest breast meat yield. Chicks obtained from the higher TM during ED 0–5 showed an increase in the female pectoralis minor’s relative weight and the pectoralis major’s relative weight in male broilers. This is when the embryonic myoblast developed [119]. Because the satellite cell’s DNA concentration, width, and diameter significantly increase at higher temperatures from cells incubated at lower temperatures (33 and 35 °C). The creatine kinase levels, myogenin expression, and myogenic determination factor 1 of satellite cells increase linearly with increasing temperature from 33 to 43 °C [91].

On d 13, the heat-treated group demonstrated a significant decrease in myofiber diameter compared to the control group. Moreover, the TM group drastically reduced satellite cell expansion [108]. The impaired muscle growth due to prolonged heat stress indicated that the high temperature hindered the satellite cells’ proliferation and changed muscle hypertrophy. A significant decrease in PCNA-expressing cells was observed in the muscle tissue of the heat-treated group. TM at 39.5 °C and 65% RH for 12 h/d stimulated rapid and sustained myoblast proliferation throughout fetal-myoblast and satellite-cell proliferation phases, resulting in increased myogenic progeny reserve in the muscle and accelerated embryo muscle development with a lasting impact on post-hatch chickens [111]. Reduced T_3_ and T_4_ concentrations were observed in the heat treatment group. Thyroid hormones, particularly T_3_, are involved in the process of myoblast cell-cycle withdrawal and differentiation into myotubes [120]. These lower embryonic plasma T_3_ concentrations support increased myoblast proliferation.

A study on Peking ducks (*Anas platyrhynchos domesticus*) showed that thermal treatment in the embryonic stage has long-lasting and immediate effects on post-hatching muscle development and phenotypic changes in muscle mass [121]. However, another study on Peking ducks reported that TM (38.5 °C and 65% RH from ED 10 to 27) displayed a negative impact on muscle growth by regulating muscle hypertrophy, atrophy, and proliferation-related genes (particularly *PAX3*) by increasing ERS [122]. The *PAX3* gene provides instructions for making a transcription factor that belongs to the *PAX* family. The PAX3 protein is a transcription factor with a C-terminal transcriptional activation domain and an N-terminal DNA binding domain with a paired box and homeodomain. This protein regulates target gene expression, affecting proliferation, survival, differentiation, and motility in various pathways. This gene is expressed during the development of skeletal muscle, the central nervous system, and neural crest derivatives [123].

TM can significantly upregulate the gene expression related to muscle growth, which can help cell proliferation by aiding the growth of satellite cells and myofiber diameter. Muscle growth also helps in regulating proper metabolism. TM appeared beneficial for early muscle development stages after hatching, which determines the final body weight in the birds. As breast muscle is an essential part of the broiler from a consumer perspective, TM showed a higher growth rate in pectoralis major and pectoralis minor muscle in the breast part. Thus, TM could increase total meat production without requiring more feed or slaughter of birds to meet market demand [118].

### Effects of TM on carcass quality

The pricing determination is influenced by carcass quality, which is accountable for achieving consumer expectations. It enables the farmer to detect the quality of the animals they are producing and, as a result, to enhance and better prepare for high-quality animals and carcasses. It can also be recognized by authorities to certify carcasses for class, quality, and condition. It also aids the meat processing industry in selecting various grades based on market and consumer needs. The phrase "carcass evaluation" refers to all the criteria determining the carcass’s average value per unit weight [124]. Evaluation of carcass quality in broiler typically includes the weight of wings, thighs, frame, fat pad, pectoralis major, and pectoralis minor muscle [110] TM at 38.8 °C during ED 7–10 and ED10–13 for 24 h/d significantly increased carcass weight (g), drip loss (%) and redness in the breast muscle. However, carcass yield (%), grill loss (%), and lightness were significantly lower than in the control group. The higher sample size weight and an accelerated pH decrease could reduce water retention capacity, leading to higher drip loss. In the same study, TM at 36.8 °C during ED 7–10 and ED10–13 for 24 h/d significantly increased grill loss (%) but significantly decreased leg yield (%) and shear force compared to the control group [92]. The analysis’s heating process might cause the increased grill loss. TM at 39 ºC at ED 7–11 for 18 h/d resulted in significantly increased skinned carcass weight (g), whereas TM at 39 °C at ED 7–18 for 18 h/d significantly decreased skinned carcass weight (g) compared to the control group [118]. It was due to significantly higher and lower BW in these two groups than the control group, respectively. In quail, TM at 41 °C at ED 6–8 and ED 12–14 for 3 h/d significantly decreased carcass weight (%) (without blood, feather, head, and shank) and carcass weight (%) (without viscera), breast weight (%) and breast muscle weight (%) than the control group. In the same study, TM at 41 °C at ED 6–8, TM at 41 ℃ at ED 12–14, and TM at 41 °C at ED 6–8 and ED 12–14 for 3 h/d significantly decreased breast muscle pH than the control [125]. The pH value is a crucial factor in determining the shelf life, with lower pH values having a significant impact. The term "more acidic" refers to a higher level of acidity, which, in turn, results in a longer shelf life for the product [126]. TM promoted the growth of the pectoral muscles in the early stages while not causing any notable changes in the characteristics of the breast meat [21]. Commercial broiler breeder eggs were incubated in trays positioned in the bottom (38.09 °C), middle (38.24 °C), and top (37.99 °C) portion of the incubator. Broilers from the bottom trays had significantly higher tender breast weight and a significantly higher proportion of breast meat (25.00%) than the middle tray’s broiler (24.54%). Nevertheless, broilers from middle trays exhibited a higher carcass yield than those from cooler top trays [98]. Prolonged exposure of eggs to high temperatures typically leads to deficiencies in embryonic development and substantial disruptions in carbohydrate and lipid metabolism. The embryo’s inability to fully utilize the nutrients from the yolk leads to the restricted and impaired growth of the embryo, resulting in subpar performance during the subsequent growth phase [127]. TM at 39.5 °C at ED 7–16 for 12 h/d significantly increased pectoralis minor (%) and yellowness of the carcass. However, it significantly decreased the cold carcass weight (g), fat pad (%), white stripe, and wooden breast [110]. It is possible that the higher temperatures during incubation in TM led to an increase in the growth of satellite cells, which may have contributed to improvements in breast muscle myopathy. The severity of myopathy and the extent of muscle degeneration significantly affect meat quality and processing characteristics [128]. However, Compared to the 0 and 3 h treatments, the 12 h incubation treatment resulted in boilers with lower pectoralis major weights. The pectoralis major weights of early hatch broilers were greater than those of the mid- and late-hatch groups. Compared to the control, the 12 h incubation treatment reduced the incidence of broilers with mild to severe myopathic characteristics [129]. It might be influenced by factors such as diet, post-hatch processing, environmental interaction, or other stimuli.

TM affects thermotolerance and can modify carcass composition by increasing the yield of breast portions and meat quality during processing. The cooking loss, carcass weight, meat pH, and moisture parameters are crucial indicators of the quality of processed meat and play a significant role in determining the juiciness and texture of cooked meat. Intermittent embryonic TM could be utilized to achieve the desired profitable outcomes of carcass quality in broilers. Considering all this evidence, TM is a very effective strategy for more carcass yield.

### Effects of TM on organ weight

Organ weight is related to the body’s nutritional efficiency, metabolic activity, energy, and amino acid requirements [130]. It is reported that eggs exposed to TM daily at 38.5 °C and 65% RH for 18 h on ED 12–18 significantly increased heart and liver weight. However, it has significantly decreased gizzard weight and colon length than control (37.8 °C throughout the incubation period) [114]. Mild heat exposure at an early age accelerates satellite cell myogenesis through specific local growth factor expression [131]. This might be the reason for the increased organ weight in this study. TM at 39 °C for 6 h/d during ED 0–5, both males and females, showed an increased heart mass compared to control chicks, implying that pumping blood to the tissues needed more effort, presumably due to inefficiencies in gas exchange. Even though the treatments did not affect lung mass, TM has affected lung morphology [132]. This implies that more energy was needed to circulate blood to the tissues, potentially because of inefficiency in gas exchange, as ventilation was reduced in both females (significantly) and males (tendency). For lung morphology, this metabolic disorder is strongly linked to pulmonary hypertension and right ventricle hypertrophy [96]. Post-hatch day 35 showed a substantial increase in gut and liver weight in the 39 °C for 18 h with 65% RH daily during the ED 7–11 groups compared to different treatment groups. However, eggs incubated in TM (39 °C) in layer chicken significantly reduced the heart weight of the control (37 °C) group [133]. Manipulating incubation temperature in the experiment led to heart hypoplasia without impacting the weight of other assessed organs, suggesting that high incubation temperatures can have varying effects on different organs. Heart hypoplasia decreases oxygen delivery to tissues, potentially hindering overall body growth and leading to metabolic issues. Broilers hatched from eggs incubated at high temperatures had a greater occurrence of ascites during the growing period than those incubated at normal temperatures [96]. TM at 39 °C at ED 7–11 for 18 h/d significantly increased gut and liver weight [118]. It might be due to the significantly higher BW and skinned carcass weight in this treatment group than in the control. TM at 39.5 °C and 65% RH for 12 h/d from ED 7 to 16 significantly decreased heart weight [87]. This could be associated with decreased levels of thyroid hormones and increased thermotolerance [79]. The hemodynamic changes likely led to increased heat loss through the skin, contributing to decreased heart mass [134]. Thus, the decrease in lower heart growth can be attributed to either a decrease in the development of cardiac cells or a reduction in glycogen but an increase in lactate concentration. In ostrich, TM (subjected to 40 ± 1 °C for 3 h/d) significantly decreased the heart and liver weight, and there was no significant change in the gizzard and proventriculus weight [135]. High setter and hatcher temperatures could reduce heart size [136]. Several common issues in poultry, like sudden death syndrome and ascites, are linked to cardiovascular system development and function problems. This decrease in liver weight could be caused by increased fat absorption from the yolk sac and activation of fat metabolism (lipolysis) for energy production. Also, this phenomenon could be attributed to the stimulation of hepatic enzyme systems responsible for glycogen breakdown (glucogenesis).

Organ weight is essential for overall growth and performance in broiler chickens. Post-hatching ontogenetic development in birds is marked by the selective growth of the heart, intestines, and liver [137]. Embryonic TM increases organ weight in the majority of instances. More studies should be focused on that to evaluate the effects of TM on organ weight and related health benefits.

### Effects of TM on blood biochemistry

Blood biochemistry can inform much about what is happening within the body. Cells release essential enzymes that help maintain blood glucose, pH, Na^+^ , and K^+^ levels. Blood biochemistry impresses when the ambient temperature rises over the thermo-neutral zone, the acid–base balance deteriorates [138], the hematocrit drops, blood volume increases [22], and lipid peroxidation increases [139]. Eggs incubated at 37.8 °C (Control) throughout the incubation period and exposed to heating at 38.5 °C 6 h/d from ED 10 to 18 (HS) of incubation have no effect of heat adaptation on the blood HCO3^−^, Na^+^ , or K^+^ concentration levels of newly hatched chicks. Also, pH was significantly higher on the hatch day from HS than in the control chicks. However, in the internal pipping and hatch stage, substantial changes in blood gases and ions occurred without affecting the blood pH level. Blood, pO_2_, Na^+^ levels, and plasma triglyceride concentrations were significantly increased at hatch. However, pCO_2_, HCO3^−^, and K^+^ levels were much lower. In addition, the levels of triglycerides and T_3_ undergo reversible changes from hatch to d 7 of incubation. These modifications indicate an embryo’s adaptive response [140]. 38.5 °C From ED 10 to 18, 6 h/d of incubation at a high temperature caused a consistent reduction of post-hatch plasma T_3_ and corticosterone changes. At d 21, regardless of parental age, the glucose level was significantly higher, and the triglyceride level was significantly lower in the high-temperature group. These changes in blood metabolites and hormones may enhance the thermoregulatory capabilities of broilers after hatching when subjected to daily high temperatures [141]. It is shown that plasma malondialdehyde and blood glucose levels of thermally challenged birds were significantly higher at an initial temperature of 35 °C (maintained for 4 d and then progressively decreased) than those of the other treatments (35 °C for the first 2 d, 35 °C for the first 7 d and 35 °C for the first 10 d). Even though the birds had a varied brooding temperature program during their first 10 d (post-hatch), there was no significant difference in blood triglyceride, creatine kinase, or uric acid levels. So, TM can enhance performance early and prevent broiler chickens from severe heat stress when they reach market age [142]. Between ED 16–18.5, a consistent 3 °C increment in incubation temperature from the standard 37.6 °C resulted in reduced thyroid hormone levels and liver glycogen deposits. This decreases blood glucose accessibility during the hatching phase, hyperglycemia, or both; plasma triglyceride and non-esterified fatty acid concentrations are reduced. It illustrates that broiler embryos are more sensitive to high incubation temperatures than low ones [127]. TM at 39.5 °C for 3 h/d during ED 16–18 increased chick thermotolerance acquisition, significantly reduced corticosterone levels, and the lowest T_3_ to T_4_ ratio in chicks exposed to 41 °C for 6 h/d thermal conditioning (TC) at d 3. During TC, significant hyperthermia was seen in control chicks, associated with greater plasma T_3_ levels [27]. The results indicate that applying TM at 39.5 °C later in embryogenesis successfully decreased stress levels in chicks exposed to stressful environmental conditions. In a study conducted on ostrich, it was found that total plasma protein, albumin, and globulin abundances were considerably lower in the TM group (38.5 °C and 45% RH for 3 h/d on d 35–37 of incubation) than in the control group (32 ± 1 °C) throughout late embryonic development. The TM group had substantially higher uric acid and creatinine concentrations than the control group. These results suggest that the administration of TM at 38.5 °C during late embryogenesis effectively mitigated the detrimental effects of heat stress on chicks exposed to strenuous environmental conditions during the early stages of development [135]. A study in the layer showed that incubating eggs at 39 °C decreases the amount of blood-ionized calcium available for bone mineralization during embryo development. It suggested that temperature management is critical during the incubation of viable layer eggs to ensure high chick quality [133].

Blood concentration (glucose, uric acid, creatinine, pH, Na^+^ , and K^+^ levels) is essential for broiler birds’ thermoregulation. Imbalanced blood concentration can trigger the stress response, which harms the body. TM may regulate blood biochemistry at an optimum level, which can help to reduce the stress response at the cellular level.

### Effects of TM on oxidative damage

Oxidative stress is the imbalance that develops when the number of reactive oxygen species (ROS) in an animal cell exceeds its antioxidant capacity. Unconfined radicals are produced in vast quantities as an inevitable by-product of numerous metabolic processes, and in some instances, such as in activated neutrophils, they are produced purposely. In addition, the body can produce free radicals in response to electromagnetic radiation from the surroundings, and they can also be obtained directly as oxidizing contaminants such as ozone and nitrogen dioxide. Damage can develop in various tissues if antioxidant defences are lacking [143]. Oxidative stress, a significant consequence of heat stress, is a crucial predictor of performance and physiologic degradation in broilers [144]. Due to oxidative damage, broiler meat loses its appearance, flavor, and nutritional value [145]. Several genes, including NADPH oxidase 4 (*NOX4*), superoxide dismutase 2 (*SOD2*), and catalase, regulate cellular homeostasis to prevent oxidative stress [146].

TM at 39 °C and 65% RH from ED 10 to 18 for 18 h/d may have an excellent long-term effect on broiler antioxidant potential. On post-hatch day (PD) 29, TM significantly decreases catalase mRNA levels in the liver but not in the spleen [147]. However, after 1 (PD 29) and 7 (PD 35) days of heat stress, the catalase level was considerably lower in TM chicks than in controls. In contrast, after 3 (PD 31), 5 (PD 33), and 7 (PD 35) days of heat stress, the *NOX4* mRNA level in TM chicks was considerably lower than in controls. After 3 (PD 31), 5 (PD 33), and 7 (PD 35) days of heat stress, *SOD2* levels in TM chicks were considerably lower than in controls [147]. One of the primary producers of ROS has been identified as the multicomponent enzyme NADPH oxidase (*NOX*). Much research has been done recently on *NOX4*, one of the seven members of the NOX family (*NOX1, NOX2, NOX3, NOX4, NOX5, DUOX1*, and *DUOX2*). Hydrogen peroxide (H_2_O_2_) is produced constitutively due to its distinctive structural characteristics. As a crucial oxygen sensor, *NOX4*-derived H_2_O_2_ has a variety of functions in cell division, migration, and death [148]. Superoxide dismutase (*SOD*) proteins help eliminate ROS by catalyzing the disproportionation of O_2_ and H_2_O_2_, followed by converting hydrogen peroxide to water by catalase or glutathione peroxidase. The vast oxidation–reduction and electron transport events in mitochondria produce oxygen radicals, which are scavenged by the mitochondrial matrix enzyme *SOD2* (*OMIM*, *147460*) [149]. In the case of TM at 39 °C and 65% RH for 18 h/d from ED 10 to 18 and acute heat stress on the post-hatch d 28 at 40 °C, *NOX4, GPX2*, *SOD2*, and catalase mRNA expression was considerably lower in TM chickens than in controls. However, avian uncoupling protein (*AvUCP*) mRNA expression was significantly increased. Also, overall antioxidant capacity, superoxidase dismutase, and catalase activity in the TM group were considerably lower than in the control group. Lowering system genes related to heat-induced oxidative stress implies that TM has a lasting influence on the development of thermotolerance in chickens [150]. In par with TM, acute heat stress (34 °C for 6, 12, and 18 h/d) also enhanced mitochondrial superoxide generation in the pectoralis muscle of chickens. This quick increase in mitochondrial substrate oxidation led to increased superoxide generation, which may be beyond AvUCP’s ability to regulate superoxide production. Down-regulation of AvUCP, on the other hand, induced increased mitochondrial superoxide generation during the latter phases of heat stress, even when substrate oxidation had reverted to normal levels [151]. Despite being present in numerous bird species, the biological function of the avian homolog of mammalian uncoupling proteins remains debatable [152]. Uncoupling protein (UCP) homologs from plants and animals may have evolved from a proton-anion transporter ancestor to form a subfamily of mitochondrial carriers. The cold environment activates UCP homologs in plants, which may affect chilling tolerance. The biochemical actions and biological capabilities of the mammalian UCP2 and UCP3 are not fully understood. The UCPs may also play a role in cellular metabolism’s reaction to an abundance of substrates by controlling ATP levels, the NAD^+^/NADH ratio, and a variety of metabolic pathways, as well as limiting superoxide generation [153].

Cold TM (36.6 °C for 6 h/d during ED 10–18) shows higher brain catalase levels in younger breeder embryos than in older ones. This encasement of the antioxidative status of TM chicks from a younger breeder can be advantageous in tissue protection because of oxidative stress caused by the colder growth conditions temperature [154]. This TM may cause changes in antioxidant routes by increasing catalase activity but decreasing AvUCP3 at the hatching day. However, an increase in the expression of the transcription factor peroxisome proliferator-activated receptor-ɣ coactivator-1α was found to promote a long-lasting rise in the expression of AvUCP3. These repetitively cold incubation settings resulted in prolonged alterations in antioxidant pathways, which might improve the health of chickens raised in cold environments [155].

The effect of TM against oxidative damage is well documented in broiler chickens. Oxidative genes act as a primary defense mechanism in the body to protect it from the heat stress response. Upregulations of these genes mean the body is in optimum condition to cope with heat stress. These studies prove that TM birds can have a higher oxidative gene expression than the control birds. Therefore, TM birds can scavenge free radicals more efficiently and reduce the negative effect of heat stress in the body.

### Effects of TM on transcriptomics

The performance of broiler chickens has now achieved its current level because of long-term genetic improvement studies [156]. However, because of this development, broilers have become more sensitive to environmental influences, and their ability to adjust to harsh conditions has weakened. Heat stress is particularly unfavorable from a commercial standpoint, as even a 1 °C increase is sufficient to reduce the productivity of most animal species [157]. Protective genotypes should be generated to combat heat stress through selective breeding, or the capacity of poultry to withstand current thermal stress should be enhanced [158]. Temperature and humidity above the ideal circumstances are implemented in the pre-hatch stage to acclimatize the broilers to heat stress. This technique, also known as ‘epigenetic adaptation’, is based on lifelong irreversible alterations in the physiological control systems of the organism generated by numerous environmental manipulations [159].

TM at 39 °C and 65% RH for 18 h/d during ED 10–18 improve the heat resistance by increasing the liver and splenic mRNA expression of *IL-6* (it may aid in regulating the process of tissue protection during the heat stress) and by regulating the expression of gene components of its production routes during subsequent acute heat stress 40 °C for 7 h. TM increased *IL-6*, *HSF3*, and *HSP70* basal mRNA expression but reduced *TLR4* basal expression levels. TM improved the expression kinetics of *HSP70, HSF3, IL-6, IL-1, TNF-α, TLR2, TLR4, NFkB50,* and *NFkB65* during heat stress [160]. *NFkB* regulates multiple aspects of innate and adaptive immunological processes and is crucial in mediating inflammatory responses. *NFkB* controls inflammasomes and stimulates the expression of numerous pro-inflammatory genes, such as those that produce cytokines and chemokines. Additionally, *NFkB* is essential for regulating the survival, activation, and differentiation of innate immune cells and inflammatory T cells. Therefore, abnormal *NFkB* activation contributes to the pathogenesis of numerous inflammatory diseases [161].

TM treatments (38.5, 39, 39.5, and 40 °C for 18 h/d, ED 12–18) significantly alter the basal mRNA expression of *HSP108, HSP90, HSF1,* and *HSF2* genes in muscle tissue on post-hatch d 14 and 28 [162]. TM at 40.5 °C for 3 h on ED 15, 16, and 17 reduced the *HSP27, HSP60, HSP70, HSP90 alpha, HSP90 beta*, and ubiquitin. A higher methylation level is observed in the promoter region of the *HSP70, HSP90 alpha*, and *beta* genes in the TM group at 42 d of age, which might be an essential factor in contributing to epigenetic adaptation [163]. This evidence suggests that exposure to TM during embryonic development has both short-term and long-term effects on the expression of HSFs in broiler chickens, manifesting after TM exposure has ended. Moreover, this change was linked to enhanced thermotolerance development in broiler chickens under heat stress. However, TM (41 °C during ED 15 to 17 for 3 h/d) exhibited no significant adverse effect on the cerebral tissue. However, mild degeneration happened in the HS group. *HSPB1, HSPB5, HSPB8*, and *HSPB9* were expressed with or without the TM. All small heat shock proteins were downregulated except *HSPB9*, which was elevated in the HS group in response to TM [164]. An examination of the gene bank reveals that *HSPB9*, among the small heat shock proteins analyzed in the present research, does not contain introns. The absence of introns may expedite the quick expression of HSPs and clarify how they can be expressed despite stressors such as heat that may disrupt RNA splicing [165]. Additionally, reduced HSP levels in thermally manipulated birds may suggest decreased harm to the cellular structures [166]. Interestingly, a study conducted on Pekin ducks yielded different results. TM at 38 °C from ED 1 to 10 increases *HSP70* mRNA expression in the bursa. The increased transcription of *HSP70* mRNA in duck embryos subjected to high incubation temperatures is likely a protective measure against the higher temperature [167]. In Japanese quail, TM at 39.5 °C from ED 10 to 13 for 12 h/d had minimal effect on the regulation of gene expression in the hypothalamus. However, In females, the effects of changes in TM gene expression induced by heat challenge (post-hatch, 36 °C for 7 h)) were most noticeable, with an approximately 20-fold increase in the number of differentially expressed genes. TM may improve the gene response to stress in the quail hypothalamus and facilitate new cellular coping mechanisms under adverse conditions, as demonstrated by the identification of differentially expressed genes related to mitochondrial and heat-response functions [168].

TM significantly impacts necessary heat shock protein-related and anti-inflammatory gene expression. Heat shock-related genes initiate the stress response at the cellular level in the body. Then, it signals the anti-inflammatory genes to start the inflammatory response. According to the studies, TM can significantly upregulate the heat shock proteins-related genes, which means TM-treated birds are more efficient in stopping the stress response in the body.

### Effects of TM on metabolism

Genetic selection for the development and efficiency of meat-type chickens has enhanced metabolic rates. In contrast, selection for reproductive qualities of egg type has resulted in increased lipid metabolism [169]. The processes underlying the acquisition of thermotolerance and molecular changes in metabolic rate have remained elusive. During embryogenesis, TM has been demonstrated to reduce O_2_ intake and heart rate, implying a decreased reclining metabolic rate and modifications in the vasomotor response [170]. In male chicks, it is reported that TM at 39 °C for 6 h/d during ED 0–5 has a higher metabolic rate under hypercapnia, and there was no change in the metabolism under normoxia/normocapnia. In the case of female chicks, the hypoxic-hypometabolic response was reduced, implying that they had a slower metabolic response to hypoxia [132], which appears detrimental to life in those circumstances [171]. Hypoxia had a decreased depressing effect on metabolism in TM chickens, which may be required to maintain a robust ventilatory response to low-O_2_ settings. These chicks had a greater hypoxic ventilatory response (significant in females, with a trend in males), which could have reduced the hypometabolic response.

TM at 39.5 °C and 65% RH for 12 h/d during ED 7–16 significantly influenced thyroid hormone metabolism by lowering the muscle mRNA regulation of *DIO3*. TM enhanced *DIO2* mRNA expression in the liver while decreasing citrate synthase activity, which is vital in the Krebs cycle. In the muscle of TM groups, the phosphorylation level of p38 mitogen-activated protein kinase, which regulates the cell stress response, was higher than in controls. TM altered energy utilization and development markers in the pectoralis major muscle and the liver. It may indicate a long-lasting adaptation by restricting energy metabolism or reducing TM during incubation when faced with a heat challenge in later life [172]. An elevated ambient temperature (32 °C for one week) changes the synthesis machinery and the beginning of translation, resulting in reduced protein synthesis rates. In chickens that received glucose–arginine therapy, glucose levels were significantly greater at 32 °C compared to 22 °C, but insulinemia did not vary significantly. The muscular expression of *PGC-1α* was approximately reduced by 40% after exposure to the heat challenge (32 °C for one week). *PGC-1α* is a transcription factor that regulates energy metabolism. So, lower expression of *PGC-1α* reflects the reduced intensity of oxidative metabolism. The infusion of glucose–arginine to fed chickens resulted in increased glycemia at 32 °C than at 22 °C, with no thermally-related variation in insulinemia, indicating that insulin has a decreased potential to promote glucose absorption or that response kinetics are delayed during heat circumstances [173]. TM at 38.9 °C from ED 10.5 to 17.5 a bolus of (U-13C) glucose in the chorioallantoic fluid raises the glucose need in chicken embryos throughout perinatal development, as evidenced by a rise in glucose oxidation and a reduction in hepatic glycogen. Possibly mediated by increased gluconeogenesis from glucogenic amino acids to permit anaerobic glycolysis, increased gluconeogenesis from glucogenic amino acids to allow anaerobic glycolysis may jeopardize fruitful hatching and impair body growth [174].

Embryonic TM can significantly influence the essential hormone related to metabolism. Then, it upregulates the genes associated with various metabolic cycles in the body, even at high environmental temperatures. Therefore, TM birds sacrifice metabolism to some extent in heat stress conditions but do not impair muscle growth. Thus, it keeps the growth at an optimum level. However, the exact role of TM in metabolism is still unknown (Tables 3 and 4).

**Table 3:** The Effects of pre-hatch thermal manipulation on health and production performance in broilers

Thermal manipulation			Effects		References
**Temperature, ºC**	**Time, h/d**	**ED**	**Increase**	**Decrease**	
15	30 min	18 and 19	Hatch weight, hatchability, body weight	Mortality, ascites (%)	[100, 175, 176]
	60 min	18 and 19	Breast muscle weight (% of BW)		[176]
30	12	10–18		Hatchability	[177]
33	3	15–17		Survivability (%), cerebellum (*HSPB1,HSPB5,HSPB9*)	[164]
34.6	4	16–18	Hatch time, hatchability		[178]
	24	16–18.5		Hatchability	[127]
36	3	0–5		Male chick body mass(g)	[132]
36.6	6	10–18	Hatchability, hatch time, body lipid content (%)	Serum T_3_ (ng/mL)	[100, 155, 179]
36.7	24	0–21	Embryo mortality	Hatchability	[76]
36.8	24	7–10 and 10–13	Carcass yield (%), grill loss (%)	Body weight	[92]
36.9	6	0–8	Wing length (mm), metatarsus length (mm)	Embryo weight (mm), Tibia length (mm)	[102]
		10–18	Embryo weight (mm), face length (mm), wing length (mm), metatarsus length (mm)		[102]
**Standard **i**ncubation temperature (37.5–37.8 °C)**					
38.1	24	0–5	Plasma testosterone in males, breast muscle weight		[70]
38.2–38.4	2	18–21	Hatchability		[180]
	4	11–21	Female fat (%), meat redness	Male fat (%)	[181]
	24	18–21		Hatchability, body weight	[180]
38.3, 38.1 and 38.0	4	19, 20 and 21	Male sex ratio		[73]
38.3	24	0–21	Embryo mortality	Hatchability	[76]
38.4	4	18–20		Skin temperature after heat stress	[182]
38.5	3	16–18		*IGF1*, T_3_ and T_4_ concentration	[86, 105]
	18	12–18	Body, carcass, heart, liver weight, muscle (*HSF2, HSF1)*	Colon length, muscle (*HSP108*, *HSP90*)	[114, 162]
	12	12–18	Hatchability, ileum (*HSP70, HSP90, HSPH1, GPX*, and *TXN*), embryonic lipid metabolism, and thermotolerance	Hatch time, total VFA concentration	[71, 183]
	6	10–18	Triglycerides	Hatchability, embryo weight, glucose, liver, and heart weight	[140, 141, 184]
	6, 9 and 12	16, 17, and 18	Body weight		[25]
38.6	8	15–20	Male vocalizations, *ANO2*, *TRAAK*		[82, 185]
38.7	4	1–5	FCR		[186]
		8–12		Mortality	[186]
		14–18	FCR		[186]
	24	11–21		Salmonella counts in cecal contents	[187]
38.8	24	7–10 and 10–13	Body weight, carcass weight	Carcass yield (%),drip loss (%), grill loss (%)	[92]
38.9	24	7–21	Embryo mortality, hatchability, hatch weight, heart weight, body weight, total mortality, breast meat yield (%)		[96]
39	6	0–5		Female chick body weight	[132]
	9	12–18	*PAX7, PCNA*		[188]
	12	12–18	Body weight, *IGF1*		[188]
		13–21	Plasma T_3_ concentration		[90]
	18	12–18	Carcass, heart, liver, muscle *(HSF2, HSF1),* Body weight, myogenin, *IGF1*	Colon length, muscle (*HSP108*, *HSP90*), hatch weight, cloacal temperature	[114, 162, 188, 189]
		7–11	Hatchability, body weight, breast, thigh muscle, gut and liver weight, muscle (*IGF1, GH*), sarcomeres length		[118, 190]
		7–18	Lactate dehydrogenase (pectoral and thigh muscle)	Hatchability, body weight, thigh muscle weight	[118, 191]
		10–18	Body weight, *SGLT1, GLUT2, FABP1, CD36*, liver (*NOX*), Spleen (*Catalase, NOX, SOD*), body temperature, methylation of the *IGF1* and *GHR* promoter regions	Hatchability, body weight, average daily feed intake, oxidative stress, serum T_3_, *GHR, IGF1, IGF2* expression	[147, 189, 192, 193]
		11–15		Hatchability, body weight	[118]
		15–18		Hatchability, body weight, thigh muscle weight	[118]
	24	0–21		Body weight, feed intake, viability (%)	[194]
		13–21		Body weight (g), tibiotarsal rigidity, femoral breaking strength	[103]
39.1	16	10–20	Embryo mortality		[195]
39.5	3	8–10	Hatchability, mortality		[29]
		16–18	Mortality, Hatchability, corticosterone (ng/mL), *HSP27*, body weight, pectoralis muscle (% of BW), myofiber diameter (μm), *PCNA, PAX7*	Hatchability	[27, 29, 97, 110, 196]
		8–10 and 16–18	Hatchability, mortality		[29]
	6	16–18	Body weight, pectoralis muscle (% of BW), myofiber diameter (μm), *PCNA, PAX7*		[97]
	12	7–16	O_2_ saturation, mortality, myofiber diameter, feed intake, cold carcass weight (g)	CO_2_ partial pressure, feed intake (kg), FCR, female body weight (kg), hatchability, body weight gain, thyroxin, hatch time (h)	[21, 87, 99, 110, 197, 198]
	18	12–18	Breast muscle, liver, gizzard weight, muscle *(HSF2)*	Carcass weight	[114, 162]
	24	7–16	Plasma corticosterone concentration	Body weight	[20, 64]
39.6	6	0–8		Hatchability	[199]
		10–18		Hatchability	
	8	15–20	Social retainment, *ANO2*, *SCN5A*	Hatchability	[82, 185]
40	3	6–8	Embryo mortality, T_3_, T_4_	Hatchability, red blood cell, white blood cell, packed cell volume	[68]
	6, 9 and 12	16, 17 and 18	Body weight, T_4_		[25]
	12	10–18		Hatchability	[177]
	18	12–18	Breast muscle, liver weight, muscle (*HSF1*, *HSF2*)	Carcass weight, muscle (*HSP108*, *HSP90*)	[114, 162]
40.5	3	15–17	Live sperm (%)	*HSP70*	[200]
40.6	4	16–18	Hatch time, plasma glucose (mg/dL), lactic acid (mmol/L)	Hatchability	[178]
	24	16–18.5		Hatchability, hatch weight, T_3_, T_4_	[127]
41	3	8–10	Metatarsus length (mm)	Embryo weight	[102]
		15–17	Cerebellum (*HSPB9*)	Hatchability, survivability (%), cerebellum (*HSPB1*)	[164]
		16–18	Embryo weight, metatarsus length (mm)	Wing length (mm), tibia length (mm)	[102]

Only significant (*P* < 0.05) post-hatch effects (excluding hatchability) are considered in the table

**Table 4:** The Effects of pre-hatch thermal manipulation on health and production performance on layers, Pekin ducks and quails

Thermal manipulation			Effects		References
**Temperature**, °C	**Time**, **h/d**	**ED**	**Increase**	**Decrease**	
36.5 (layer)	6	10–18	Hatchability, hatch time, SOD(U/mL), T_3_		[201]
**Standard incubation temperature (37.5–37.8 °C)**					
38 (Pekin duck)	24	0–10	*IL6*, *IFNG*, *IL10*, *HSP70*		[167]
		11–24	Liver weight	Hatchability	[121]
38.5 (layer)	6	10–18		Hatchability, hatch time	[201]
39 (layer)	24	0–21		Egg mass, egg conductance, heart weight, BEecf (mmol/L)	[133]
39.1 (quail)	2	4–14	Hatchability	Hatch time	[202]
40 (layer)	4	14–18		Hatchability, hatch time	[28]
41 (quail)	3	6–8	Body weight, breast meat pH	Female hatch weight, abdominal fat weight (%)	[88, 125]
		12–14	Breast meat pH	Body weight	[125]
		6–8 and 12–14	Carcass weight (%), breast weight (%), breast meat pH		[125]

Only significant (*P* < 0.05) post-hatch effects (excluding hatchability) are considered in the table

## Challenges of TM in poultry

Several difficulties are involved in implementing a TM strategy, even though it has demonstrated promising outcomes in enhancing growth rates, feed conversion, and disease resistance. Among these difficulties are:

- *Precision*: Maintaining accurate temperature control is critical, particularly during embryonic development, when the temperature is essential for the embryo’s healthy development. Developmental problems or death may come from temperatures too high or too low. Following the age and particular needs of the birds, which can change depending on the breed, sex, and production system, the temperature should also be adjusted. Also, the TM starting and ending day is very important. Very early stages of TM can kill the embryo or hamper post-hatch growth and performance. If TM begins at a later age, that might not affect the embryo; thus, there will be no performance improvement in later life.
- *Environmental factors*: Environmental elements like altitude, humidity, and ventilation can also impact how well TM works. Low ventilation can raise the risk of respiratory disease, while high humidity can make cooling methods less efficient. It is crucial to consider the humidity rate during the development period to prevent the dehydration of embryos. To maximize the advantages of TM, the production environment should be adequately monitored and maintained.
- *Genetics and breed*: The birds’ genetics and breed can impact the efficiency of TM. Genetic selection can affect how a breed reacts to temperature manipulation, and certain breeds may respond to TM more effectively than others. Consequently, it is essential to consider the birds’ genetics and breed selection when using TM.
- *Cost*: Specialized tools are needed for TM, including incubators, hatchers, and brooders, which can be costly to buy and operate. Furthermore, the energy usage and upkeep expenses related to heating and cooling systems might be high.
- *Labor*: Temperature and humidity levels must be regularly checked and adjusted during the TM. This method can be labor- and time-intensive in larger enterprises where multiple incubators, hatchers, and brooders may be needed.
- *Training and education*: It is crucial to acknowledge that hatcheries can utilize TM to enhance long-term on-farm chicken thermoregulation. This necessitates a strong collaboration between hatchery practitioners and other professionals. The cost of implementing TM in hatcheries should be weighed against the benefits it brings to other practitioners. Proper training and education are essential for successfully implementing TM. The appropriate administration of the equipment, monitoring, and adjusting temperature and humidity levels should all be covered in staff training. This can help to limit the likelihood of errors and guarantee that the birds experience the tremendous benefits of TM.
- *Health and safety*: Using heating and cooling systems has some inherent risks, including fire risk, electrical concerns, and producing poisonous chemicals. In addition, stress from touching the chicks during the process may negatively impact the birds’ health and welfare. Proper administration, maintenance, and equipment inspection are essential to maintain the workers’ and birds’ health and safety.
- *Ethics*: According to some critics, the well-being of the birds may be jeopardized if TM is used to enhance performance and speed up growth. When using this approach, it is essential to consider the risks involved, such as the likelihood that skeletal and metabolic diseases will become an issue depending on the type of TM applied. For ethical grounds, specific customers and animal welfare organizations may also object to TM.
- *Regulatory requirements*: Depending on the region, regulations governing TM in chicken production may exist. Hence, manufacturers should know the legal requirements before using TM and secure the required licenses and approvals.

## The potential of TM in poultry

Recent research has indicated that the TM approach may have advantages for poultry production. Exposure to various temperatures during specific developmental phases can significantly affect a bird’s growth rate, feed conversion efficiency, immune system, heat tolerance, behavior, and egg production.

Reduced resource utilization is one way that TM can support sustainability. Birds need less feed to grow to the same size because of increased feed conversion efficiency, which can result in lower feed costs and less demand for feed supplies. This can lessen the impact of chicken production on the environment and conserve natural resources. Improved animal well-being is a potential side effect of TM for sustainability. Birds may be more able to handle environmental challenges and have higher overall health and well-being if aggressiveness decreases and heat tolerance increases [83, 84]. This may lessen the need for veterinary interventions and administering antibiotics and other drugs, which may harm the environment.

TM can also reduce greenhouse gas emissions from poultry production. We have evidence that thermally manipulated birds hatched 5–6 h earlier than the customarily incubated birds. It can reduce energy consumption in industries. Thus, cutting the production cost means the prices will be more affordable. Birds create less waste and use fewer resources to produce the same amount of meat or eggs by increasing feed conversion efficiency. In terms of greenhouse gas emissions and fertilizer runoff, this can help lessen the environmental impact of chicken production. Finally, TM can help create a more sustainable food system by improving the effectiveness of resource utilization in chicken production.

TM in poultry production has the potential to advance sustainability by lowering resource consumption, enhancing animal welfare, reducing greenhouse gas emissions, and improving resource efficiency. TM should support the birds’ health and well-being while minimizing environmental effects; as with any production method, it is crucial to assess the potential repercussions and trade-offs carefully.

## Conclusion

**Fig. 5:**
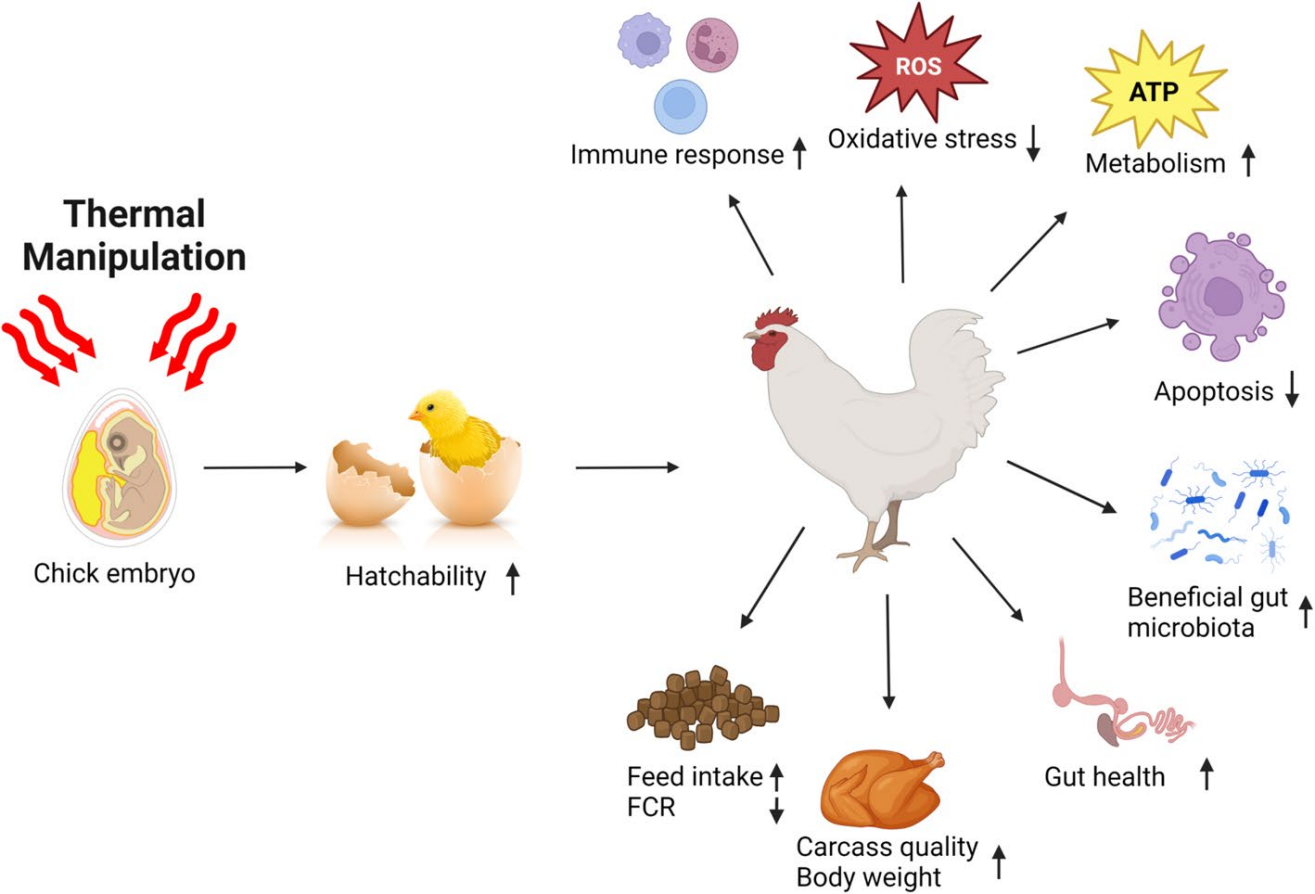
Effects of thermal manipulation (TM) in broiler chicken (Created by Biorender.com)

The manipulation of thermal conditions during the embryonic development of broiler chickens is a promising technique that can enhance hatch parameters and post-hatch growth performance. A review of the available studies reveals that adjusting the temperature at specific stages of embryonic development can significantly improve hatchability, body weight, feed conversion ratio, and growth rate. However, the optimal temperature ranges and durations of thermal manipulation may vary depending on several variables, such as breed, environmental circumstances, and management techniques. Based on the available evidence, a temperature range of 38.5–39.5 °C for 12–18 h/d during mid to late embryonic stages shows promising results. Nonetheless, additional research is necessary to refine thermal manipulation techniques and gain a deeper understanding of the underlying physiological principles. Overall, these findings have significant implications for the poultry industry. These studies indicate that thermal manipulation can be an effective way to enhance both the hatchability and growth performance of broiler chickens. However, more research is necessary to optimize this technique and determine its practicality on a larger scale Fig. 5.

## Abbreviations

AvUCP: Avian uncoupling protein
BW: Body weight
CRH: Corticotropin-releasing hormone
DUOX1: Dual oxidase 1
DUOX2: Dual oxidase 2
DNA: Deoxyribonucleic acid
DP: Differential peaks
DIO2: Deiodinase enzymes 2
DIO3: Deiodinase enzymes 3
EA: Early heat treatment
ED: Embryonic days
EST: Eggshell temperature
FCR: Feed conversion ratio
GPX2: Glutathione peroxidase 2
GH: Growth hormone
HS: Heat stress
HSP: Heat shock protein
HSF1: Heat shock factor 1
HSF2: Heat shock factor 2
HSF3: Heat shock factor 3
HSF4: Heat shock factor 4
HSPB1: Heat shock protein B1
HSP60: Heat shock protein 60
HSP70: Heat shock protein 70
HSP90: Heat shock protein 90
HSP100: Heat shock protein 100
HSPH1: Heat shock protein H1
HSPB1: Heat shock protein B1
HSPB5: Heat shock protein B5
HSPB8: Heat shock protein B8
HSPB9: Heat shock protein B9
HPT-axis: Hypothalamic-pituitary-thyroid axis
HPA-axis: Hypothalamic-pituitary-adrenal axis
IT: Incubation treatment
IL-1: Interleukin 1
IL-6: Interleukin 6
IGF-1: Insulin-like growth factor 1
LA: Late heat treatment
MyoD: Myoblast determination protein 1
NADPH: Nicotinamide adenine dinucleotide phosphate
NOX1: NADPH oxidase 1
NOX2: NADPH oxidase 2
NOX3: NADPH oxidase 3
NOX4: NADPH oxidase 4
NOX5: NADPH oxidase 5
NT: Natural treatment
NFkB: Nuclear factor kappa
NFkB50: Nuclear factor kappa 50
NFkB65: Nuclear factor kappa 65
PAX3: Paired box gene 3
PAX7: Paired box gene 7
PCNA: Proliferating cell nuclear antigen
PD: Post-hatch day
PGC-1α: Peroxisome proliferator-activated receptor-gamma coactivator 1α
PO/AH: Preoptic anterior hypothalamic
PVN: Paraventricular nucleus
RH: Relative humidity
ROS: Reactive oxygen species
RNS: Reactive nitrogen species
SOD1: Superoxide dismutase 1
SOD2: Superoxide dismutase 2
SRIH: Somatotropin release-inhibiting hormone
TSH: Thyroid stimulating hormone
TC: Thermal conditioning
TLR2: Toll-like receptor 2
TLR4: Toll-like receptor 4
TM: Thermal manipulation
TRH: Thyrotropin-releasing hormone
UCP: Uncoupling protein
UCP2: Uncoupling protein 2
UCP3: Uncoupling protein 3
